# Introducing our 20th anniversary collection

**DOI:** 10.1186/s13075-019-2049-x

**Published:** 2019-11-20

**Authors:** Christopher D Buckley, Harris Perlman

**Affiliations:** 1 Rheumatology Research Group, Institute for Translational Inflammation Research, College of Medical and Dental Sciences, University of Birmingham Research Laboratories, Queen Elizabeth Hospital Birmingham, Birmingham, B15 2WD UK; 2 Kennedy Institute of Rheumatology, University of Oxford, Roosevelt Drive, Oxford, Headington UK; 3 Department of Medicine, Division of Rheumatology, Feinberg School of Medicine, Northwestern University, 240 E. Huron Street, McGaw M338, Chicago, IL 60611 USA

For our 20-year anniversary, we have chosen to celebrate by commissioning seven editorials that look back at seven original articles that we have published over the last two decades. These articles are among the most highly cited, downloaded, and referenced original articles that the journal has published. We were delighted that all the original lead authors agreed to take up our challenge to look back, with hindsight at the way in which their articles have become so impactful. We wanted to let our authors be our advocates, and indeed, they have done so, in a humble yet striking way.

For example, in March 2005, *Arthritis Research and Therapy* published “Systemic lupus erythematosus induced by anti-tumour necrosis factor alpha therapy: a French national survey” [1]. As the lead author Michel De Bandt confesses, he thought “The subject seemed quite banal at the time,” but in fact, it was the first set of observations that anti-TNF drugs can cause drug-induced lupus. ANA and anti-DNA are now routinely, before patients start starting an anti-TNF mainly on the basis of this publication.

Over 12 years ago, we published a paper on the use of the interleukin-1 (IL1) inhibitor anakinra in acute gout [2]. It was a pilot study comprising an extended case series and animal data to validate the concept that IL1 played a pivotal role in the initiation of MSU crystal-induced inflammation. As Alexander So recollects, he chose to publish in *Arthritis Research and Therapy* because it publishes proof-of-concept studies rapidly, in comparison with conventional journals. The finding that anakinra, was effective to treat gout proof that the inflammasome (intracellular “machine” that processes pro-IL1 to mature and secreted IL1) is responsible for pathogenesis in gout. Anakinra is now used to treat patients who are unable to receive or have contraindications to conventional drugs used to treat acute gout.

A study led by Karim Raza published in 2005 [3] identified that people with rheumatoid arthritis (RA) assessed, within 3 months of symptoms onset, had a synovial fluid cytokine profile that was distinct from that of patients with other inflammatory arthritides of similarly short duration. This profile, which was transient, was characterized by cytokines of stromal and T cell origin. These findings suggested that the first few months after symptom onset were associated with changes in the early RA joint that differed from those operating at later stages. The implications from these findings was later disease is not more of early disease have provided the rationale for some of the ongoing early prevention studies in RA.

In 2008, we published “Cardiovascular (CV) in patients with rheumatoid arthritis: results from the QUEST-RA Study” [4]. As Antonio Naranjo and Iván Ferraz-Amaro reflect, the QUEST-RA study has opened new research avenues in a field that has become essential to the care of patients with RA.

The QUEST-RA study’s finding that treatments for RA reduced cardiovascular risk in patients with RA was fundamental to understanding the relationship between systemic inflammation and CV disease. This has proven to be a crucial result, not only for patients with inflammatory arthritis, but also for the field of CV disease in healthy populations.

When anti-TNF was first licensed for RA, physicians were cautious regarding the new biologics and possible cancer development. Thus, a key unanswered question remained. What was the background rate of malignancies in patients with RA? Was the occurrence of malignancies in patients with RA different from the GP? In 2008, we published the answer [5]. As Teresa A. Simon describes, “our extensive literature meta-analysis review showed no difference in overall malignancy rates between the RA population and the GP.”

A decade has passed since Désirée van der Heijde and Robert Landewé published their comparison of the effect of adalimumab with data from a historic cohort on the progression of structural damage in the spine of patients with ankylosing spondylitis (AS) [6]. The somewhat unusual comparator group was formed by a historic cohort of patients with AS treated with non-steroidal anti-inflammatory drugs (NSAIDs) and conventional synthetic disease-modifying anti-rheumatic drugs (csDMARDs). No effect could be observed, and there is still no definite proof that TNF inhibitors (TNFi) inhibit spinal structural damage. This uncoupling of inflammation from damage has proven with time to be a very important biological finding.

We the final word to Annette van der Helm-van Mil whose study in 2005, “Antibodies to citrullinated proteins and differences in clinical progression of rheumatoid arthritis,” was one of the first to link citrullination with disease progression in RA [7]. She wisely reflects, “In general, studies that change the field are those that either improve the understanding of disease pathogenesis or have an impact on patients care in daily practice. Such important studies are relatively easy recognized in retrospect, but are less easy recognizable at the time of first reporting, because subsequent studies are generally required to validate or expand on the initial findings.”

## Data Availability

Not applicable

## Abbreviations

ANA: Antinuclear antibodies
AS: Ankylosing spondylitis
csDMARDs: Conventional synthetic disease-modifying anti-rheumatic drugs
CV: Cardiovascular disease
IL1: Interleukin-1
GP: General population
MSU: Monosodium urate
NSAIDs: Non-steroidal anti-inflammatory drugs
RA: Rheumatoid arthritis
TNF: Tumor necrosis factor
TNFi: Tumor necrosis factor inhibitors
